# Granulocyte Colony-Stimulating Factor Does Not Influence *Clostridium Perfringens* α-Toxin-Induced Myonecrosis in Mice

**DOI:** 10.3390/toxins11090509

**Published:** 2019-08-30

**Authors:** Masaya Takehara, Yuuta Sonobe, Hiroto Bandou, Keiko Kobayashi, Masahiro Nagahama

**Affiliations:** Department of Microbiology, Faculty of Pharmaceutical Sciences, Tokushima Bunri University, Yamashiro-cho, Tokushima 770-8514, Japan

**Keywords:** Clostridial myonecrosis, phospholipase C, host-pathogen interaction, granulocyte colony-stimulating factor

## Abstract

*Clostridium perfringens* type A causes gas gangrene characterized by myonecrosis and development of an effective therapy for treating affected patients is of clinical importance. It was recently reported that the expression of granulocyte colony-stimulating factor (G-CSF) is greatly up-regulated by *C. perfringens* infection. However, the role of G-CSF in *C. perfringens*-mediated myonecrosis is still unclear. Here, we assessed the destructive changes in *C. perfringens*-infected skeletal muscles and tested whether inhibition of G-CSF receptor (G-CSFR) signaling or administration of recombinant G-CSF affects the tissue injury. Severe edema, contraction of muscle fiber diameter, and increased plasma creatine kinase activity were observed in mice intramuscularly injected with *C. perfringens* type A, and the destructive changes were α-toxin-dependent, indicating that infection induces the destruction of skeletal muscle in an α-toxin-dependent manner. G-CSF plays important roles in the protection of tissue against damage and in the regeneration of injured tissue. However, administration of a neutralizing antibody against G-CSFR had no profound impact on the destructive changes to skeletal muscle. Moreover, administration of recombinant human G-CSF, filgrastim, imparted no inhibitory effect against the destructive changes caused by *C. perfringens*. Together, these results indicate that G-CSF is not beneficial for treating *C. perfringens* α-toxin-mediated myonecrosis, but highlight the importance of revealing the mechanism by which *C. perfringens* negates the protective effects of G-CSF in skeletal muscle.

## 1. Introduction

*Clostridium perfringens* type A is a Gram-positive anaerobic bacterium that causes gas gangrene [1,2]. The disease is characterized by myonecrosis, shock, multiple organ failure, and death of patients [3]. The infection progresses so rapidly that death precedes diagnosis in some patients and, thus, the development of an effective therapy for treating patients with *C. perfringens*-mediated myonecrosis is of utmost importance.

The well-known virulence factors produced by *C. perfringens* type A are α-toxin (or phospholipase C), which has both phospholipase C (PLC) and sphingomyelinase (SMase) activities [3,4], and θ-toxin (or perfringlysin O), which is a pore-forming and cholesterol-dependent cytolysin [5,6]. Using a mouse model of *C. perfringens*-mediated myonecrosis, it was reported that α-toxin-deficient and θ-toxin-deficient strains delay the spread of muscle necrosis [7]. θ-toxin is cytotoxic to polymorphonuclear leukocytes and macrophages [8,9,10]. α-Toxin and θ-toxin enhance intravascular cell aggregation, leading to vascular occlusion, and impair the host immune response by impeding inflammatory cell infiltration to the site of the infection, which is a hallmark of clostridial myonecrosis [11,12,13,14,15]. Additionally, we have reported that α-toxin interferes with the production of neutrophils by inhibiting their differentiation in bone marrow, which is also related to the impairment of innate host immunity [16,17]. Furthermore, *C. perfringens* produces other toxins and enzymes, including a collagenase, hyaluronicdase, sialidases, and the cysteine protease α-clostripain [5,18]. Thus, various toxins involved in *C. perfringens*-mediated myonecrosis have been identified and the infection process has become increasingly clear. However, understanding of the recovery process remains limited.

Low et al. recently analyzed the host transcriptome from *C. peringens*-infected muscle and found that expression of *Csf3*, the gene encoding granulocyte colony-stimulating factor (G-CSF), is greatly up-regulated by the infection [19]. G-CSF is a cytokine that stimulates the mobilization of hematopoietic stem cells and the production of neutrophils and exerts its effects by binding to G-CSF receptor (G-CSFR) [20]. G-CSFR is not only expressed on hematopoietic cells, but also non-hematopoietic cells, including oval cells, myoblasts, cardiomyocytes, and neural tissue [21,22,23,24]. G-CSF was reported to be involved in the development of cardiac muscle and skeletal muscle by promoting their cell proliferation [23,25]. Moreover, a growing body of scientific evidence has indicated that treatment with G-CSF ameliorates tissue injury in various organs, as follows: A neuroprotective effect of G-CSF in cerebral ischemia was reported [26]; G-CSF improves outcomes in mouse models of amyotrophic lateral sclerosis and doxorubicin-induced cardiomyopathy [27,28]; anti-apoptotic effects of G-CSF were observed on neurons [26,29]; G-CSF enhances muscle cell proliferation and strength following muscle injury in rats [30]; and G-CSF promotes liver repair by enhancing oval cell proliferation [21]. Thus, G-CSF plays important roles in protecting tissue against damage and in regenerating injured tissue. However, it is still unknown how the amplified production of G-CSF following *C. perfringens* infection affects the progression of *C. perfringens*-mediated myonecrosis. Here, we assessed the destructive changes in *C. perfringens*-infected skeletal muscles and tested whether inhibition of G-CSFR signaling or administration of recombinant G-CSF affects the tissue injury.

## 2. Results

### 2.1. C. perfringens Induces Myonecrosis in a Toxin-Dependent Manner

**Figure 1:**
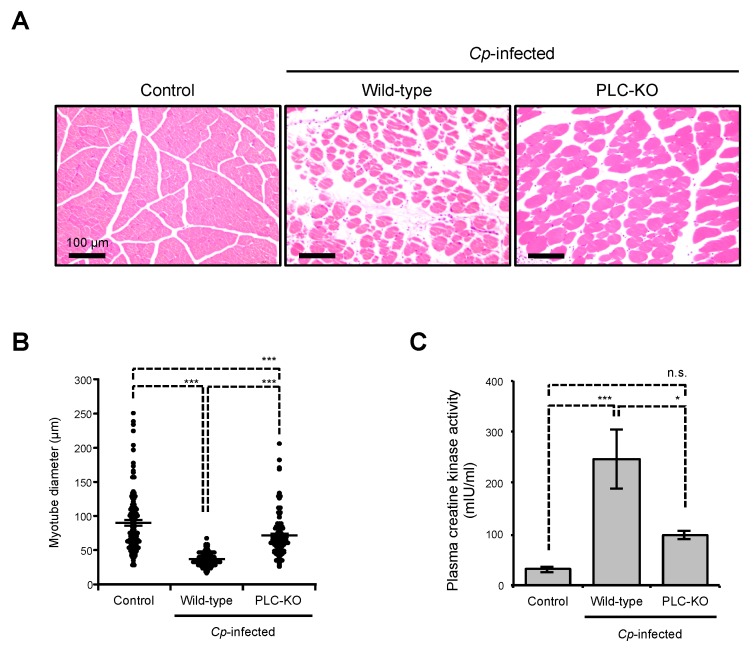
Destructive changes in *C. perfringens*-infected skeletal muscle. Mice were intramuscularly injected with 1 × 10^7^ CFU of *C. perfringens* Strain 13 (Wild-type), PLC-KO (PLC-KO), or TGY (tryptone, glucose, and yeast extract) medium as a control (Control). (**A**) Muscles were isolated 24 h after infection. Hematoxylin and eosin (H&E)-stained sections are shown. Representative H&E-stained sections of three independent experiments are shown. (**B**) The diameters of at least 100 muscle fibers of three independent experiments were measured. (**C**) Peripheral blood was isolated 24 h after infection and plasma creatine kinase activities were determined using a creatine kinase activity assay kit (*n* = 8 per condition). One-way ANOVA was employed to assess significance. Values are the mean ± standard error. * *p* < 0.05; *** *p* < 0.001; n.s., not significant.

It was previously reported that *C. perfringens* infection causes myonecrosis in mice and the severity of skeletal muscle necrosis decreased in mice injected with the α-toxin-deficient strain [7]. At 24 h post-infection, severe edema and a contraction of muscle fiber diameter were observed in mice intramuscularly injected with wild-type (WT) *C. perfringens* type A (Figure 1A,B). The release of creatine kinase, which is a marker of muscle damage, into the circulation following *C. perfringens* infection or inoculation of α-toxin has been reported [31]. As shown in Figure 1C, plasma creatine kinase activity increased in *C. perfringens*-infected mice. These results indicate that *C. perfringens* infection induces the destruction of skeletal muscle. The destructive changes observed in a *plc* gene-knockout mutant of *C. perfringens* (PLC-KO)-infected mice were lower than those in mice injected with WT *C. perfringens* (Figure 1A–C), demonstrating that *C. perfringens* induces myonecrosis in an α-toxin-dependent manner.

### 2.2. Inhibition of G-CSFR Does Not Affect C. perfringens α-Toxin-Induced Myonecrosis In Vivo

**Figure 2:**
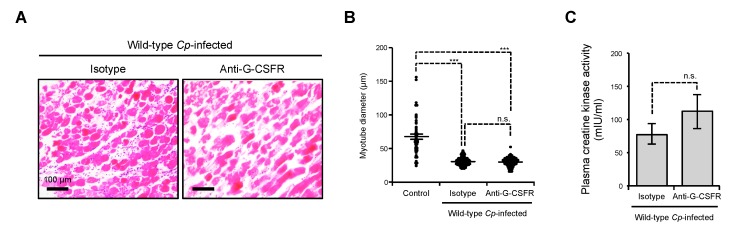
Neutralization of G-CSFR does not influence *C. perfringens* α-toxin-induced myonecrosis. Mice were intramuscularly injected with 1 × 10^7^ CFU of *C. perfringens* Strain 13 (Wild-type Cp-infected) or TGY medium as a control (Control). To neutralize G-CSFR, a specific antibody against mouse G-CSFR (Anti-G-CSFR) or an isotype control antibody (Isotype) was intraperitoneally administered to the *C. perfringens*-infected mice shortly after the injection of *C. perfringens*. (**A**) Representative H&E-stained sections of three independent experiments are shown. (**B**) The diameters of at least 100 muscle fibers of three independent experiments were measured. (**C**) Plasma creatine kinase activities were determined using a creatine kinase activity assay kit (*n* = 11 per condition). One-way ANOVA or the two-tailed Student’s *t*-test was employed to assess significance. Values are the mean ± standard error. *** *p* < 0.001; n.s., not significant.

The augmented production of G-CSF in *C. perfringens*-infected skeletal muscle was observed in two independent experiments [19,32], but the role of G-CSF in *C. perfringens*-induced myonecrosis has not been elucidated. To reveal how the amplified production of G-CSF affects the progression of *C. perfringens*-mediated myonecrosis, we treated *C. perfringens*-infected mice with a neutralizing antibody against G-CSFR shortly after the injection of *C. perfringens* and assessed the destructive changes in skeletal muscles at 24 h after infection. The dose of the antibody was 10 times higher than that in the previous report [33]. Many studies have indicated that treatment with G-CSF ameliorates tissue injury in various organs, such as skeletal muscle, brain, and cardiac muscle [26,27,28,29,30]. Therefore, we expected that the administration of a neutralizing antibody against G-CSFR exacerbates the destructive changes, i.e., severe edema, contraction of muscle fiber diameter, and release of creatine kinase. Contrary to our expectation, the administration of the neutralizing antibody had no profound impact on the destructive changes (Figure 2A–C). Thus, inhibition of G-CSFR signaling did not affect *C. perfringens* α-toxin-induced myonecrosis in vivo.

### 2.3. Filgrastim Has no Protective Effect on Skeletal Muscle During C. perfringens α-Toxin-Mediated Myonecrosis

**Figure 3:**
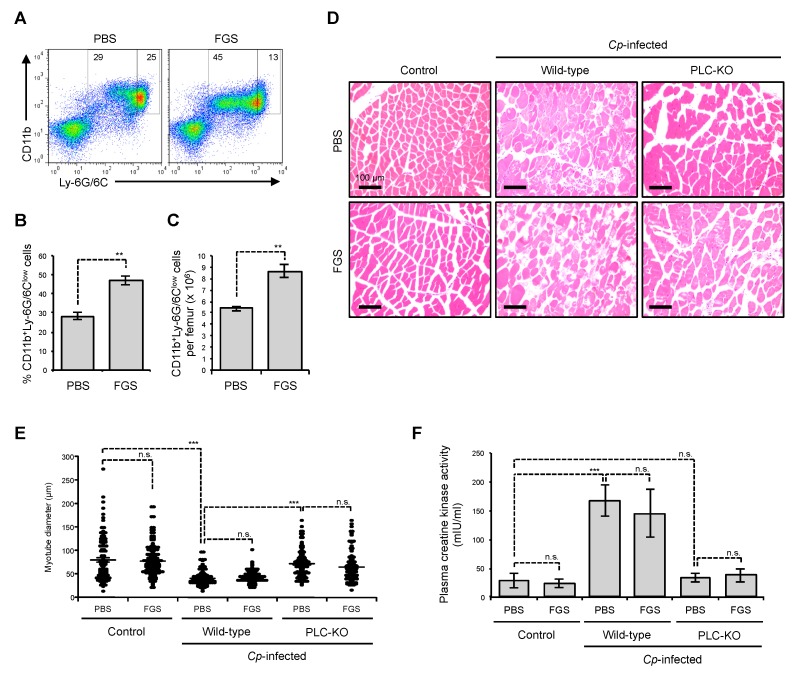
Filgrastim has no protective effect against *C. perfringens* infection in skeletal muscle. (**A**–**C**) Mice were subcutaneously injected with 1.5 µg of filgrastim (FGS) or the same volume of phosphate buffered saline (PBS), bone marrow cells (BMCs) were isolated after 24 h, and flow cytometry analysis was performed. A representative flow cytometry profile of three independent experiments (**A**) and the proportion (**B**, *n* = 3) and absolute number (**C**, *n* = 3) of CD11b^+^Ly-6G/6C^low^ immature neutrophils are shown. (**D**–**F**) Mice were intramuscularly injected with 1 × 10^7^ CFU of *C. perfringens* Strain 13 (Wild-type), PLC-KO (PLC-KO), or TGY medium as a control (Control). Shortly after the injection, 1.5 µg of filgrastim (FGS) or the same volume of PBS was administered subcutaneously and muscles were isolated 24 h after infection. (**D**) Representative H&E-stained sections of three independent experiments are shown. (**E**) The diameters of at least 100 muscle fibers of three independent experiments were measured. (**F**) Plasma creatine kinase activities were determined using a creatine kinase activity assay kit (*n* = 5–8). One-way ANOVA or the two-tailed Student’s *t*-test was employed to assess significance. Values are the mean ± standard error. ** *p* < 0.01;*** *p* < 0.001; n.s., not significant.

G-CSF represents antiapoptotic effects on neurons [26,29]. Additionally, G-CSF was reported to promote skeletal muscle regeneration and development by stimulating myoblast proliferation [25]. In the clinical setting, recombinant G-CSF is widely used to treat neutropenia caused by cancer treatment, to stimulate production of neutrophils [34]. A Neuroprotective effect of recombinant human G-CSF in transient focal ischemia of mice has been reported [26], suggesting that human G-CSF and murine G-CSF show biological cross reactivity. To test whether treatment with G-CSF is beneficial for *C. perfringens* α-toxin-mediated myonecrosis, we administered recombinant human G-CSF, filgrastim, to *C. perfringens*-infected mice and assessed the destructive changes. As shown in Figure 3A–C, administration of filgrastim increased the proportion and number of CD11b^+^Ly-6G/6C^low^ immature neutrophils in bone marrow at 24 h after the injection, demonstrating that the injection protocol is appropriate for assessing the efficacy of filgrastim. However, filgrastim had no profound impact on the severe edema and contraction of muscle fiber diameter at 24 h after infection with *C. perfringens* (Figure 3D,E). Moreover, the administration did not diminish the release of creatine kinase (Figure 3F). Together, our results suggest that filgrastim has no protective effect on skeletal muscle during *C. perfringens* α-toxin-mediated myonecrosis.

## 3. Discussion

In the present study, we showed that *C. perfringens* induces myonecrosis in an α-toxin dependent manner, but PLC-KO infection still induces the destruction of skeletal muscle. Using a mouse model of *C. perfringens*-mediated myonecrosis, it was reported that θ-toxin-deficient strains delay the spread of muscle necrosis [7], meaning that θ-toxin is also involved in *C. perfringens*-mediated myonecrosis. Furthermore, *C. perfringens* produces other toxins and enzymes including a collagenase, hyaluronicdase, sialidases, and the cysteine protease α-clostripain [5,18]. Thus, various toxins involved in *C. perfringens*-mediated myonecrosis have been identified. Therefore, myonecrosis caused by PLC-KO infection could be explained by the existence of the other toxins.

A growing body of scientific evidence indicates that treatment with G-CSF ameliorates tissue injury in various organs [26,27,28,29,30]. However, our results demonstrated that G-CSF has no influence on *C. perfringens*-induced myonecrosis in mice. In the present study, we evaluated the destructive changes at 24 h after *C. perfringens* infection. Low et al. reported that *C. perfringens*-mediated clostridial myonecrosis progresses extremely rapidly; early signs of the disease in mouse skeletal muscle are observed from two hours after infection [19]. We previously reported that secretion of G-CSF starts to be up-regulated more than three hours after stimulation of toll-like receptor 2 by its agonist, peptidoglycan [35]. It is thus possible that tissue injury by *C. perfringens* infection commences before the increased production of G-CSF.

In the present study, *C. perfringens*-infected mice were administrated with the G-CSFR antibody or recombinant G-CSF shortly after the infection for the following reasons. One is that G-CSFR-expressing cells are absent in non-injured skeletal muscle while G-CSFR is clearly expressed in injured skeletal muscle [25], and then we thought that pre-treatment with the G-CSFR antibody or recombinant G-CSF does not represent any effect on skeletal muscle. Moreover, pre-treatment might increase the chance of detecting off-target effects for the same reason. The second is that pre-treatment with G-CSFR antibody or recombinant G-CSF should affect granulopoiesis and change the total number of neutrophils in the whole body. Neutrophils have been shown to be involved in the elimination of injected *C. perfringens* in skeletal muscle [16], so the change of the number of neutrophils could influence the survival of *C. perfringens*. The difference of the survival probably affects destructive changes by *C. perfringens* infection. Thus, it would be difficult to evaluate the results of pre-treatment experiments. However, it would still be worth testing if pre-treatment with G-CSFR antibody or G-CSF prior to *C. perfringens* infection improves the outcome of *C. perfringens* infection.

Recently, we reported that α-toxin augmented the production of G-CSF from endothelial cells only in the presence of TLR2 agonists, suggesting that α-toxin enhances Toll-like receptor 2 (TLR2) signaling [32]. During *C. perfringens* infection, bacterial components stimulate TLR2. The increased G-CSF could act on myeloid progenitors to promote granulopoiesis, which is one of the host defense mechanisms against pathogenic bacterial infection, but α-toxin disturbs G-CSF-mediated granulopoiesis by reducing the expression of G-CSFR on neutrophils [32]. Thus, *C. perfringens* overwhelm the host defense by producing α-toxin. G-CSF represents antiapoptotic effects on various tissues including skeletal muscle, brain, and cardiac muscle [26,27,28,29,30], which is another host defense mechanism, but no protective effect by G-CSF treatment was observed during *C. perfringens* infection in the present study. Further investigation is necessary to elucidate the detailed mechanism, but it is possible that α-toxin is involved in the negation of the tissue protective effect of G-CSF on skeletal muscles.

*C. perfringens* type A is known to produce several toxins related to the destruction of skeletal muscle. α-Toxin, which is a cytotoxic bacterial phospholipase C, plays a key role in the pathogenesis of myonecrosis [3,4]. In the steady state, G-CSFR is expressed in proliferating mouse myoblasts, C2C12 cells, differentiated C2C12 myotubes, and human and mouse primary skeletal muscle [22]. However, we recently reported that α-toxin desensitizes neutrophils to G-CSF by inducing the degradation of G-CSFR [32]. Additionally, *C. perfringens* type A produces θ-toxin, which is a pore-forming cholesterol-dependent cytolysin [5,6]. Muscle tissue from mice infected with a θ-toxin-deficient strain displays a delayed spread of muscle necrosis [7]. Thus, cytotoxic bacterial toxins produced by *C. perfringens* type A are involved in the pathogenesis of clostridial myonecrosis. It is possible that these toxins disturb and impair the antiapoptotic effects of G-CSF, or that treatment with G-CSF has no protective effect against muscle cell necrosis. Furthermore, G-CSFR expression increases in muscle cells after injury by injection of cardiotoxin [25], suggesting that damage to skeletal muscles makes the muscle cells more sensitive to G-CSF. Further studies are needed to clarify whether and how the increased G-CSF production affects skeletal muscle regeneration during the recovery period.

## 4. Conclusions

In conclusion, our results suggest that G-CSF is not beneficial for the treatment of *C. perfringens* α-toxin-mediated myonecrosis, but they emphasize the importance of revealing the mechanism by which *C. perfringens* negates the protective effects of G-CSF in skeletal muscle. We hope that further studies will unveil this mechanism and that our results contribute to the development of new therapeutic strategies for treating patients with clostridial myonecrosis.

## 5. Materials and Methods

### 5.1. Mice

For all experiments, mice aged more than 8 weeks old were used. The animal experiments were approved by the Animal Care and Use Committee of Tokushima Bunri University and procedures were performed in accordance with institutional guidelines (Approval code: 17-3 Date: 1 April 2018). C57BL/6J mice were from SLC (Shizuoka, Japan).

### 5.2. Reagents and Strains

Fluorescein isothiocyanate (FITC)- and phycoerythrin (PE)-conjugated specific antibodies against mouse CD11b (clone M1/70) or Ly-6G (clone 1A8) and purified rat anti-mouse CD16/CD32 (Fc Block) were purchased from BD Biosciences (San Jose, CA, USA). Recombinant human G-CSF, filgrastim, was from Mochida Pharmaceutical Co., Ltd. (Tokyo, Japan). All other chemicals were of the highest grade available from commercial sources. *C. perfringens* wild-type (WT) Strain 13 was used in this study. The preparation of a *plc* gene-knockout mutant of *C. perfringens* (PLC-KO) was as described in our previous report [16].

### 5.3. Flow Cytometry Analysis

Mice were subcutaneously injected with 1.5 µg of filgrastim or the same volume of PBS and bone marrow cells (BMCs) were isolated after 24 h, as previously described [16]. The antibodies described above were used to label cells after blocking Fc-receptors with purified rat anti-mouse CD16/CD32. Antibodies were diluted with PBS containing 2% fetal bovine serum (FBS; AusGeneX, Molendinar, QLD, Australia). The labeled cells were analyzed using a Guava easyCyte (Millipore, Billerica, MA, USA). FlowJo software (Tree Star, Ashland, OR, USA) was used to analyze the data.

### 5.4. Bacterial Culture and Infection

Bacterial culture and infection were performed as previously described [16]. Briefly, *C. perfringens* WT strain 13 or PLC-KO were grown in TGY (tryptone, glucose, and yeast extract) medium under anaerobic conditions at 37 °C, and exponentially-growing bacteria were harvested, washed, and re-suspended in TGY medium. The bacteria were injected into the femoral muscles of the mice. Residual bacteria were serially diluted, plated on brain heart infusion agar plates, and cultured anaerobically at 37 °C to quantify the colony-forming units (CFU).

### 5.5. Myotube Morphology Analysis

*C. perfringens*-infected muscles were isolated 24 h after infection. The isolated tissues were fixed in 4% paraformaldehyde and embedded in paraffin. Paraffin sections were cut from the tissue and stained with hematoxylin and eosin to visualize muscle fibers. Pictures of the muscle fibers were taken using a digital camera and the diameters of muscle fibers were measured using a DS-L4 (Nikon, Tokyo, Japan). The diameters of at least 100 muscle fibers were measured for each condition.

### 5.6. Administration of G-CSF and Inhibition of G-CSFR In Vivo

Mice were subcutaneously injected with 1.5 µg of filgrastim or the same volume of PBS, shortly after the injection of *C. perfringens*. To inhibit G-CSFR, a neutralizing antibody against G-CSFR (R&D systems, Minneapolis, MN, USA) was used as described in a previous report [33]. The neutralizing antibody or Rat IgG_2B_ isotype control (R&D systems, Minneapolis, MN, USA) was intraperitoneally administered to *C. perfringens*-infected mice at 5 µg per mouse shortly after the injection of *C. perfringens*. This dose was 10 times higher than that in the previous report [33].

### 5.7. Measurement of Plasma Creatine Kinase

Using heparinized syringes, peripheral blood was obtained via the vena cava from mice 24 h after *C. perfringens* injection. To assess plasma creatine kinase activity, a commercial creatine kinase activity assay kit (Abcam, Cambridge, MA, USA) was used, and measurements were performed in accordance with the manufacturer’s instructions.

### 5.8. Statistical Analysis

All statistical analyses were performed with Easy R (Version 1.38, Saitama Medical Center, Jichi Medical University, Saitama, Saitama, Japan, 2018) [36]. Differences between the two groups were evaluated using the two-tailed Student’s *t*-test. One-way analysis of variance (ANOVA) followed by Tukey’s test was used to evaluate differences among three or more groups. Differences were considered to be significant for values of *p* < 0.05.
